# Differential viral load of surgical masks worn by patients infected with respiratory viruses

**DOI:** 10.1017/ash.2024.312

**Published:** 2024-09-16

**Authors:** Shuk Ching Wong, Vincent Chi-Chung Cheng

**Affiliations:** Queen Mary Hospital, The University of Hong Kong

## Abstract

**Background:** This study investigated viral load in surgical masks worn by adult patients infected with respiratory viruses. **Method:** Surgical masks were dissected into eight pieces at pre-selected sites: inner, middle, and outer layers at the nose (N1-N3) and mouth (M1-M3), as well as full-thickness on the right (RS) and left (LS) sides. Viral load was detected, correlated with nasopharyngeal specimens and patients' demographics. **Result:** Among 230 patients infected with influenza A virus (n=91), respiratory syncytial virus (RSV) (n=61), and SARS-CoV-2 (n=78) from April 1 to August 31, 2023, 90.9% (209/230) were from the medical specialty. Of the 230 surgical masks collected, viral RNA was detected in 79.6% at one or more sites, with 75.7% positive at N1 or M1, 55.2% positive at N3 or M3, and 22.6% exhibiting viral RNA at all sites. Pearson correlation showed viral load correlation between nasopharyngeal specimens and N1 (0.244, p=0.002) and M1 (0.174, p=0.031). The mean viral load at N1 (4.14 ± 1.46 log10 copies/ml) was significantly higher than M1 (3.74 ± 1.32 log10 copies/ml, p=0.014) and N3 (3.58 ± 1.27 log10 copies/ml, p=0.003). Significant differences in viral load were observed across N1-N3 and M1-M3 in RSV patients, but not in influenza A or SARS-CoV-2 patients. SARS-CoV-2 patients exhibited significantly lower viral load at RS and LS sites compared to influenza A or RSV patients. **Conclusion:** Viral RNA was detected in N3 or M3 sites in our masks, highlighting the potential risk associated in these areas. Differential viral load across various sites in surgical masks worn by patients infected with different respiratory viruses warrants further investigation.

